# Development and validation of a prediction model for unemployment and work disability among 55 950 Dutch workers

**DOI:** 10.1093/eurpub/ckac045

**Published:** 2022-05-25

**Authors:** Patricia Ots, Karen M Oude Hengel, Alex Burdorf, Suzan J W Robroek, Daan Nieboer, Jolinda L D Schram, Sander K R van Zon, Sandra Brouwer

**Affiliations:** Department of Health Sciences, Community & Occupational Medicine, University of Groningen, University Medical Center Groningen, Groningen, The Netherlands; Department of Public Health, Erasmus University Medical Center, Rotterdam, The Netherlands; Department of Work Health Technology, Netherlands Organization for Applied Scientific Research, TNO, Leiden, The Netherlands; Department of Public Health, Erasmus University Medical Center, Rotterdam, The Netherlands; Department of Public Health, Erasmus University Medical Center, Rotterdam, The Netherlands; Department of Public Health, Erasmus University Medical Center, Rotterdam, The Netherlands; Department of Public Health, Erasmus University Medical Center, Rotterdam, The Netherlands; Department of Health Sciences, Community & Occupational Medicine, University of Groningen, University Medical Center Groningen, Groningen, The Netherlands; Department of Health Sciences, Community & Occupational Medicine, University of Groningen, University Medical Center Groningen, Groningen, The Netherlands

## Abstract

**Background:**

This study developed prediction models for involuntary exit from paid employment through unemployment and disability benefits and examined if predictors and discriminative ability of these models differ between five common chronic diseases.

**Methods:**

Data from workers in the Lifelines Cohort Study (*n* = 55 950) were enriched with monthly information on employment status from Statistics Netherlands. Potential predictors included sociodemographic factors, chronic diseases, unhealthy behaviours and working conditions. Data were analyzed using cause-specific Cox regression analyses. Models were evaluated with the C-index and the positive and negative predictive values (PPV and NPV, respectively). The developed models were externally validated using data from the Study on Transitions in Employment, Ability and Motivation.

**Results:**

Being female, low education, depression, smoking, obesity, low development possibilities and low social support were predictors of unemployment and disability. Low meaning of work and low physical activity increased the risk for unemployment, while all chronic diseases increased the risk of disability benefits. The discriminative ability of the models of the development and validation cohort were low for unemployment (*c* = 0.62; *c* = 0.60) and disability benefits (*c* = 0.68; *c* = 0.75). After stratification for specific chronic diseases, the discriminative ability of models predicting disability benefits improved for cardiovascular disease (*c* = 0.81), chronic obstructive pulmonary disease (*c* = 0.74) and diabetes mellitus type 2 (*c* = 0.74). The PPV was low while the NPV was high for all models.

**Conclusion:**

Taking workers’ particular disease into account may contribute to an improved prediction of disability benefits, yet risk factors are better examined at the population level rather than at the individual level.

## Introduction

Preventing early involuntary exit from paid employment is important for both individuals and the society as a whole.^1^^,^^2^ Paid employment provides an individual the possibility to earn an income and perform activities that provide meaning and fulfilment and is associated with better health.^1^ From a societal perspective, entering unemployment or disability benefits leads to social costs due to less productivity, higher welfare costs and more health care utilization.^2^ In industrialized countries, retaining workers in the labour market becomes even more important in the light of an ageing population and, consequently, a higher proportion of workers with a chronic disease.^3^

A large variety of risk factors to exit paid employment involuntary have been identified at the population level. Meta-analyses showed that poor health, including having a chronic disease or self-perceived poor health, and unhealthy behaviours, such as obesity and lack of physical activity, are associated with unemployment and disability benefits.^4^^,^^5^ Regarding work-related factors, low job control, low rewards and high (physical) demands are risk factors for disability benefits.^6–9^ Low decision latitude, low work social support and high job insecurity are found to predict unemployment.^6^^,^^10^ Especially for workers with chronic diseases, increased attention to these risk factors is needed to ensure that people are able to continue their working lives.^11^

Several studies have developed prediction instruments to assess an individual’s risk of early exit from paid employment^12^ or disability benefits.^12–14^ Prediction models do not only indicate which factors are associated with an event but also estimate to what extent a specific individual has an increased risk to leave paid employment. This is useful within an occupational health context, as preventive efforts to reduce early exit from paid employment can be better targeted at high-risk groups of workers. Plomp et al. found that higher age, low education, informal caregiving, a larger social network and low self-esteem were risk factors for early exit from work within 3 years among workers 55 years and older with a chronic disease or low physical performance.^12^ Another study among soldiers showed that the number of months with temporary restrictions, frequent work excusals, high outpatient care utilization and psychotropic medication were strong predictors for receiving disability benefits over a period of 9 months.^13^ Among the general Finnish population, older age, lower socioeconomic position, smoking, self-rated poor health, a higher number of sickness absences in the previous year, chronic illnesses, sleep problems and a higher body mass index (BMI) were all predictive of disability benefits over a period of approximately 9.5 months. Within this study, an alternative prediction model showed that job strain was the only predictor for disability benefits.^14^ Previous prediction models often take frequent work excusals or sick leave into account when predicting the risk of early exit from paid employment.^13^^,^^14^ While these are strong predictors of future work disability, they are, in contrast to health behaviour and working conditions, not modifiable.

Although the described prediction models have been developed in various settings, a few concerns need to be pointed out on the predictability of exit from paid employment. First, an important methodological issue is whether the C-index is a suitable measure of model performance. The C-index for the three studies was moderate to strong, ranging from 0.70 to 0.85.^12–14^ In models where the outcome of interest has a low occurrence, the models might actually predict remaining in the labour force rather than leaving paid employment early. Therefore, it is relevant to estimate the positive and negative predictive values (PPV and NPV, respectively), which indicate to what extent prediction models are able to identify individuals at risk to leave paid employment or predict who will stay employed.^15^ Second, previous studies have examined specific exit routes into disability^13^^,^^16^ or examined exit routes together.^12^ Since unemployment and disability benefits might act as communicating vessels, it is important to construct prediction models and calculate model performance taking these competing risks into account. Third, a common critique is that most prediction models are not externally validated and, thereby, are too optimistic about performance.^17^ A clear need exists to externally validate these models, whereby the decision model’s prognostic performance, developed in one cohort, is tested in another cohort. Lastly, prediction models that focus on subgroups of workers may have a higher performance than models for the general population. As having a chronic disease is a strong predictor for leaving paid employment through disability benefits^18^^,^^19^ and as the impact on daily functioning and work differs across diseases,^20^ it is of interest to estimate prediction models for workers with a chronic disease specifically and to compare the performance of these models.

The objectives of this study are (i) to develop and externally validate prognostic prediction models for exit from paid employment through unemployment and disability benefits, (ii) to investigate if predictor effects and discriminative ability of the models differ between chronic diseases. If the prediction model is able to identify workers at risk of early exit from paid employment, then occupational health professionals could use these models to support workers, e.g. by optimizing their work environment.

## Methods

### Study design and sample

The current study used data from the Lifelines Cohort and Biobank Study^21^ as the development cohort and data from the Study on Transitions in Employment, Ability and Motivation (STREAM) for the validation cohort,^22^ and both were linked to register data of Statistics Netherlands.

Lifelines is a multi-disciplinary prospective population-based cohort study using a unique three-generation design to examine the health and health-related behaviours of 167 729 persons living in the North of The Netherlands. It employs a broad range of investigative procedures in assessing the biomedical, sociodemographic, behavioural, physical and psychological factors that contribute to the health and disease of the general population, with a special focus on multimorbidity and complex genetics. Participants were recruited between November 2006 and December 2013 through general practices, family referral and self-registration.^21^ Lifelines was conducted according to the guidelines in the Declaration of Helsinki and approved by the Medical Ethics Committee of the University Medical Center Groningen (ethics number: 2007/152).

Participants were selected if they were between 18 and 65 years and employed at wave 3 (response rate wave 3 = 62.5%; Supplementary Fig. S1). Information on health behaviours and working conditions was also retrieved from this wave. Information on sociodemographic factors and clinical measures for the classification of the included diseases was retrieved from the baseline measures. The median time between the baseline measures and wave 3 was 25 months [interquartile range (IQR) 23–29]. Lifelines data were enriched with data from Statistics Netherlands with information on main income components, social benefit pensions and gross wages derived from the Dutch tax registers and stored in the social statistical database (SSB).^23^ Data were available on a monthly basis from the time of enrolment until December 2018. The median time at risk was 54 months (IQR 44–66).

The validation cohort STREAM is a longitudinal cohort study among older workers aged 45 years and older from 2010 onwards. STREAM was also linked to monthly information on income components from SSB. For more information on the items and constructs for STREAM, see Supplementary file SB.

### Outcome variable

Involuntary exit was defined as exiting paid employment early through unemployment or disability benefits.^24^ Persons with a disability benefit received benefits for at least 50% of their income. Unemployed persons received either unemployment benefits due to losing their job or social security benefits. An individual needed to be unemployed or receiving disability benefits for at least three months to be included as an event.

### Predictors

#### Sociodemographic factors

Sociodemographic factors included age, gender, educational level and marital status. Educational level was categorized into low, medium and high educational level. Marital status was dichotomized into being in a relationship versus not being in a relationship.

#### Chronic disease and multimorbidity

At baseline, cardiovascular disease (CVD), chronic obstructive pulmonary disease (COPD), depression, rheumatoid arthritis and type 2 diabetes mellitus were classified based on previous studies conducted in Lifelines.^25^^,^^26^ Clinical measures, self-report and medication use were used to classify participants as having one of the chronic diseases. Participants with ≥2 chronic diseases were considered as having multimorbidity.

#### Working conditions

Working conditions were measured using six dimensions from an adapted version of the Copenhagen Psychosocial Questionnaire (COPSOQ II).^27^ Quantitative demands were measured with two items on getting behind in work and having enough time for work. Work pace was measured with two items on having to work very fast and having a high work pace. Influence at work was measured with items on having influence on the work one has to do and whether one has a high degree of influence on one’s work. Possibilities for development were assessed by asking whether one has the possibility to learn new things through work and whether work requires one to take initiative. Meaning of work was measured with two items asking whether someone considers their work to be important and meaningful. Social support was measured by asking about help and support from colleagues and one’s superior (two questions) and by asking how often colleagues/one’s superior are/is willing to listen to work-related problems (two questions) (α = 0.76).

All questions were scored on a five-point scale, ranging from 1 (almost never/never) to 5 (always). The answer categories of the working conditions were recoded so that a higher score reflected poorer working conditions. The domain scores were estimated as the sum of scores on the questions within each domain and were multiplied by 0.5 for social support to ensure consistency across domains. Thus, scores could range from 2 to 10 for all domains.

#### Health behaviour

Smoking was dichotomized with categories ‘non-smoking’ (including ex-smokers) and ‘smoking’. Physical activity was assessed based on one question from the ‘Short QUestionnaire to ASsess Health enhancing physical activity’ (SQUASH)^28^: ‘On average, how many days per week do you cycle, do odd jobs, garden, or exercise for a total of at least half an hour?’. Participants were asked how often they ate (fresh) fruit in the past month and how often they ate (cooked or stir-fried) vegetables. Both questions had seven response categories on an ordinal scale (‘6–7 days per week’, ‘4–5 days per week’, ‘2–3 days per week’, ‘1 day per week’, ‘2–3 days per month’, ‘1 day per month’ and ‘not during the preceding month’). BMI was based on self-reported weight. Participants were categorized as having a healthy weight (BMI ≥18.5–≤25 kg/m^2^), overweight (BMI ≥25.0–≤30 kg/m^2^) or obese (BMI ≥30.0 kg/m^2^).

### Statistical analyses

First, missing values were examined in the development cohort and ranged from 0.9% for marital status to 33.6% for fruit and vegetable intake. Missing values were imputed using the R mice-package, imputing 20 datasets based on multiple imputation by chained equations. Imputation for the working conditions was performed on item-level and domain scores were calculated after data imputation. Second, cause-specific Cox proportional hazard regression models were fitted to the m = 20 imputed datasets and pooled to analyze the effects of the predictors on early exit from paid employment through unemployment and disability benefits, taking into account competing risks. Individuals were censored in case of missing data or when they exited paid employment through (early) retirement or economic inactivity. Backward elimination was used based on the m = 20 pooled datasets. Variables with the highest *P* values were removed one by one to obtain a more parsimonious model with variables that had a significant contribution to the events. For variable selection, *P* < 0.10 was considered significant. The C-index (concordance) was examined to evaluate discriminative ability of the models. The C-index ranges from 0.5 to 1 and a higher C-index indicates better discriminative ability of the model. Third, models including interaction terms with the chronic diseases were examined and analyses were stratified for the five different chronic diseases. Stratified analyses included multimorbidity instead of specific diseases. The C-index was provided and the area under the curve (AUC) was calculated. Lastly, external model validation was performed for the final models using STREAM data. The C-index and the AUC were calculated for all final models and calibration graphs are shown. For the final models for unemployment and disability benefits, the sensitivity, specificity, PPV and NPV were calculated for different thresholds of 5, 10 and 20% as the risk of early exit varies between these values.^18^ Disease-specific models were not possible to externally validate as the sample size in the different disease subgroups became too small in STREAM. Analyses were conducted using R version 3.6.2.

## Results

### Baseline characteristics

The final study population of the development cohort consisted of 55 950 workers with a mean age of 44.4 years (SD = 9.8). The majority of the study population was female (59.7%) and in a relationship (87.6%). More workers left paid employment through unemployment (8.3%) than through disability benefits (1.7%; table 1). Workers with depression were most likely to become unemployed (13.7%) and workers with rheumatoid arthritis were most likely to receive disability benefits (5.9%; Supplementary table S1). In the validation cohort STREAM, 14.2% of workers exited into unemployment and 4.4% of workers received disability benefits during follow-up. Only the first event of early exit from paid employment was considered. However, 64.1% of workers with unemployment and 17.1% of workers with disability benefits returned to work. Attrition analyses showed that the individuals included during follow-up were slightly older [M = 45.7 (SD = 12.9) vs. M = 42.8 (SD = 13.3)], more often female (59.8% vs. 56.3%) and perceived their health as better (88.2% vs. 91.5% good health) compared to individuals who dropped out.

**Table 1:** Study characteristics of workers in development cohort (Lifelines, *n* = 55 950) and validation cohort (STREAM, *n* = 10 093)

	Lifelines—development cohort			STREAM—validation cohort		
	Paid employment (*n* = 50 368)	Unemployment (*n* = 4628)	Disability benefits (*n* = 954)	Paid employment (*n* = 8209)	Unemployment (*n* = 1435)	Disability benefits (*n* = 449)
Sociodemographic factors						
Age in years, M (SD)	44.4 (9.7)	44.6 (10.1)	42.9 (11.5)	53.8 (5.4)	53.4 (4.6)	53.8 (4.6)
Age, years, median (range)	46 (19–45)	46 (20–44)	45 (21–45)	54 (44–64)	55 (45–63)	55 (45–63)
Gender, % male	40.9	37.4	27.5	57.3	56.3	47.9
Marital status (% in a relationship)	88.0	83.7	84.2	78.8	76.4	74.2
Educational level (%)						
Low	21.4	28.8	31.1	25.3	27.6	33.6
Intermediate	42.1	40.5	43.2	38.3	39.8	42.3
High	36.5	30.7	25.8	36.5	32.6	24.1
Chronic disease (%)						
CVD	1.0	1.1	2.5	9.4	10.2	15.6
COPD	3.6	4.5	5.6	7.1	7.9	15.4
Depression	1.9	3.5	6.7	3.3	5.6	13.6
Diabetes	1.7	2.2	3.8	6.7	7.4	9.8
Rheumatoid arthritis	1.3	1.5	4.8	31.6	31.6	59.2
Multimorbidity (%)	8.8	11.7	20.5	10.4	12.4	30.0
Health behaviours						
Physical activity (days per week), M (SD)	4.2 (2.0)	4.0 (2.0)	4.0 (2.2)	4.3 (2.1)	4.1 (2.2)	4.3 (2.2)
Smoking (% current)	17.2	21.8	24.2	20.4	26.7	29.9
Fruit intake (days per week), M (SD)	5.8 (1.5)	5.7 (1.6)	5.7 (1.6)	–	–	–
Vegetable intake (days per week), M (SD)	5.7 (1.2)	5.6 (1.2)	5.7 (1.1)	–	–	–
BMI, %						
Healthy weight	49.3	47.2	47.1	35.9	33.7	29.5
Overweight	38.3	37.1	33.1	44.7	44.8	42.0
Obesity	12.4	15.7	19.8	19.4	21.5	28.6
Working conditions (sum score), M (SD)						
Quantitative demands	4.5 (1.7)	4.4 (1.8)	4.4 (1.7)	6.6 (1.6)	6.7 (1.6)	6.6 (1.8)
Work pace	6.7 (1.5)	6.6 (1.7)	6.7 (1.7)	6.0 (1.6)	6.1 (1.7)	6.1 (1.8)
Possibilities for development	4.8 (1.5)	5.2 (1.7)	5.3 (1.6)	4.2 (1.1)	4.2 (1.2)	4.3 (2.0)
Meaning of work	3.7 (1.4)	4.1 (1.6)	3.9 (1.6)	4.6 (2.0)	4.7 (2.0)	4.4 (2.1)
Influence at work	5.5 (1.7)	5.7 (1.8)	6.0 (1.8)	4.3 (1.4)	4.3 (1.4)	4.6 (1.6)
Social support	4.8 (1.4)	5.1 (1.6)	5.0 (1.6)	4.8 (1.5)	5.0 (1.6)	5.0 (1.7)

*Notes*: Higher scores reflect poorer working conditions.

– This measure was not available in STREAM; STREAM included the following chronic diseases: heart disease, respiratory disease, psychological disease, diabetes and musculoskeletal disease. The ‘paid employment’ category includes workers who are censored during follow-up.

### Risk factors for unemployment

For unemployment, backward stepwise model selection resulted in a final model including 11 variables (complete model in Supplementary table S2). Higher age, female gender and low educational level increased the risk of unemployment whereas being in a relationship decreased the risk (table 2). Furthermore, depression, smoking and obesity increased the risk while lower physical activity decreased the risk of unemployment. Low possibilities for development, low meaning of work and low social support also increased the risk of unemployment. A C-index of 0.62 [95% confidence interval (CI): 0.61–0.63] was observed.

**Table 2:** The influence of personal and work-related predictors on involuntary exit from paid employment in the development cohort (Lifelines, *n* = 55 950) and validation cohort (STREAM, *n* = 10 093)

	Lifelines—development cohort		STREAM—validation cohort	
	Unemployment (*n* = 4628) C = 0.62 (0.61–0.63)	Disability benefits (*n* = 954) C = 0.68 (0.66–0.70)	Unemployment (*n* = 1435) C = 0.60 (0.58–0.62)	Disability benefits (*n* = 449) C = 0.75 (0.73–0.77)
	*R* ^2^ = 0.02	*R* ^2^ = 0.02	*R* ^2^ = 0.02	*R* ^2^ = 0.06
	HR (95% CI)	HR (95% CI)	HR (95% CI)	HR (95% CI)
Sociodemographic factors				
Age (per 10 years)	1.04 (1.01–1.08)	0.80 (0.75–0.86)	1.50 (1.34–1.68)	1.56 (1.27–1.93)
Gender	1.21 (1.14–1.29)	1.74 (1.50–2.02)	1.07 (0.96–1.19)	1.28 (1.05–1.55)
Marital status (in a relationship)	0.74 (0.68–0.80)	0.84 (0.71–1.01)	0.95 (0.97–1.25)	0.92 (0.75–1.15)
Educational level (high = ref)				
Intermediate	1.03 (0.96–1.11)	1.24 (1.06 1.46)	1.10 (0.97–1.25)	1.41 (1.11–1.80)
Low	1.29 (1.19–1.41)	1.73 (1.43–2.10)	1.14 (1.00–1.32)	1.58 (1.22–2.04)
Chronic disease				
CVD		2.64 (1.74–4.00)		1.59 (1.22–2.06)
COPD		1.43 (1.07–1.90)		1.80 (1.39–2.34)
Depression	1.43 (1.22–1.67)	2.51 (1.94–3.25)	1.51 (1.20–1.89)	3.20 (2.43–4.22)
Diabetes		1.89 (1.34–2.68)		1.20 (0.86–1.66)
Rheumatoid arthritis		3.05 (2.26–4.11)		2.54 (2.09–3.08)
Health behaviours				
Physical activity (0–7 days)	0.98 (0.96–0.99)		0.97 (0.95–1.00)	
Smoking	1.26 (1.17–1.35)	1.35 (1.16–1.57)	1.32 (1.17–1.49)	1.50 (1.22–1.84)
Fruit intake (high = ref)				
Intermediate			n/a	n/a
Low			n/a	n/a
Vegetable intake (high = ref)				
Intermediate			n/a	n/a
Low			n/a	n/a
BMI (healthy weight = ref)				
Overweight	1.01 (0.95–1.08)	0.96 (0.83–1.11)	1.06 (0.94–1.20)	1.09 (0.87–1.37)
Obese	1.21 (1.11–1.32)	1.39 (1.16–1.66)	1.14 (0.99–1.32)	1.35 (1.04–1.75)
Working conditions				
Quantitative demands				
Work pace				
Possibilities for development	1.06 (1.04–1.09)	1.07 (1.02–1.13)	1.02 (0.97–1.07)	1.03 (0.95–1.12)
Meaning of work	1.12 (1.09–1.14)		1.03 (1.00–1.05)	
Influence at work		1.04 (1.00–1.09)		1.09 (1.02–1.16)
Social support	1.11 (1.09–1.14)	1.07 (1.02–1.12)	1.11 (1.08–1.15)	1.04 (0.98–1.11)

*Notes*: *n* reflects number of workers who exit paid employment through this route; higher scores reflect poorer working conditions.

n/a indicates that this measure was not available in STREAM; STREAM included the following chronic diseases: heart disease, respiratory disease, psychological disease, diabetes and musculoskeletal disease; HR, hazard ratio.

### Risk factors for disability benefits

With regard to disability benefits, 13 variables remained in the final model (complete model in Supplementary table S2). Female workers and workers with a low or intermediate educational level had an increased risk of disability benefits (table 2). A lower age was associated with a higher risk to receive disability benefits. Being in a relationship was associated with a lower risk. All five chronic diseases were associated with an increased risk. Furthermore, smoking and obesity increased the risk. Finally, low possibilities for development, low influence and low social support increased the risk of disability benefits. A C-index of 0.68 (95% CI: 0.66–0.70) was found.

### Disease-specific models for disability benefits

For unemployment, the interaction between predictors and depression was significant (Supplementary table S3). For disability benefits, the interaction between predictors and CVD, COPD and rheumatoid arthritis was significant (table 3). The C-index improved in models for disability benefits for CVD (C = 0.81, 95% CI: 0.71–0.91), COPD (C = 0.74, 95% CI: 0.68–0.80) and diabetes (C = 0.74, 95% CI: 0.66–0.82). The AUC values retrieved when applying the final models to workers with CVD, COPD, depression, rheumatoid arthritis and diabetes at 24 months of follow-up were 0.58 (SE = 0.05), 0.62 (SE = 0.06), 0.57 (SE = 0.03), 0.64 (SE = 0.04) and 0.65 (SE = 0.04), respectively.

**Table 3:** The influence of personal and work-related predictors on disability benefits within disease groups in the development cohort (Lifelines, *n* = 55 950)

	CVD (*n* = 583)	COPD (*n* = 2085)	Depression (*n* = 1188)	Rheumatoid arthritis (*n* = 988)	Diabetes (*n* = 775)
	C = 0.81 (0.71–0.91)	C = 0.74 (0.68–0.80)	C = 0.61 (0.53–0.69)	C = 0.69 (0.61–0.77)	C = 0.74 (0.66–0.82)
	*R* ^2^ = 0.14	*R* ^2^ = 0.05	*R* ^2^ = 0.02	*R* ^2^ = 0.07	*R* ^2^ = 0.08
	HR (95% CI)	HR (95% CI)	HR (95% CI)	HR (95% CI)	HR (95% CI)
Sociodemographic factors					
Age (per 10 years)	4.52 (1.74–11.72)	2.38 (1.37–4.16)	0.89 (0.67–1.19)	1.04 (0.69–1.55)	1.12 (0.67–1.89)
Gender	1.64 (0.69–3.90)	0.79 (0.45–1.38)	1.03 (0.59–1.81)	1.40 (0.71–2.76)	1.74 (0.86–3.53)
Marital status (in a relationship)	4.66 (0.59–36.71)	2.29 (0.71–7.36)	1.25 (0.63–2.48)	0.95 (0.33–2.71)	1.63 (0.57–4.71)
Educational level (high = ref)					
Intermediate	1.67 (0.31–8.95)	2.43 (0.81–7.29)	1.02 (0.47–2.22)	0.51 (0.22–1.16)	1.49 (0.48–4.68)
Low	4.55 (0.95–21.79)	3.34 (1.12–9.92)	1.66 (0.76–3.61)	1.05 (0.47–2.36)	1.67 (0.51–5.45)
Multimorbidity	2.29 (0.98–5.34)	1.33 (0.64–2.79)	1.16 (0.56–2.41)	0.85 (0.39–1.86)	2.31 (1.14–4.68)
Health behaviours					
Physical activity (0–7 days)					
Smoking	3.52 (1.36–9.10)	1.69 (0.95–3.03)	1.46 (0.86–2.47)	1.76 (0.91–3.40)	2.03 (0.95–4.34)
Fruit intake (high = ref)					
Intermediate					
Low					
Vegetable intake (high = ref)					
Intermediate					
Low					
BMI (healthy weight = ref)					
Overweight	1.54 (0.42–5.70)	0.55 (0.28–1.07)	0.96 (0.53–1.71)	2.41 (1.05–5.55)	0.79 (0.27–2.37)
Obese	2.27 (0.60–8.61)	1.18 (0.58–2.40)	1.24 (0.66–2.34)	3.78 (1.61–8.88)	1.75 (0.66–4.67)
Working conditions (higher is worse)					
Quantitative demands					
Work pace					
Possibilities for development	0.99 (0.75–1.31)	1.06 (0.87–1.31)	1.05 (0.88–1.27)	1.14 (0.91–1.43)	0.84 (0.64–1.09)
Meaning of work					
Influence at work	1.06 (0.84–1.33)	1.20 (1.01–1.41)	1.10 (0.94–1.28)	0.87 (0.72–1.05)	1.27 (1.02–1.59)
Social support	1.07 (0.82–1.40)	1.06 (0.88–1.27)	1.04 (0.88–1.22)	1.06 (0.86–1.31)	1.18 (0.94–1.48)

*Notes: n* reflects number of workers who exit paid employment through this route; higher scores reflect poorer working conditions.

### External model validation

**Figure 1:**
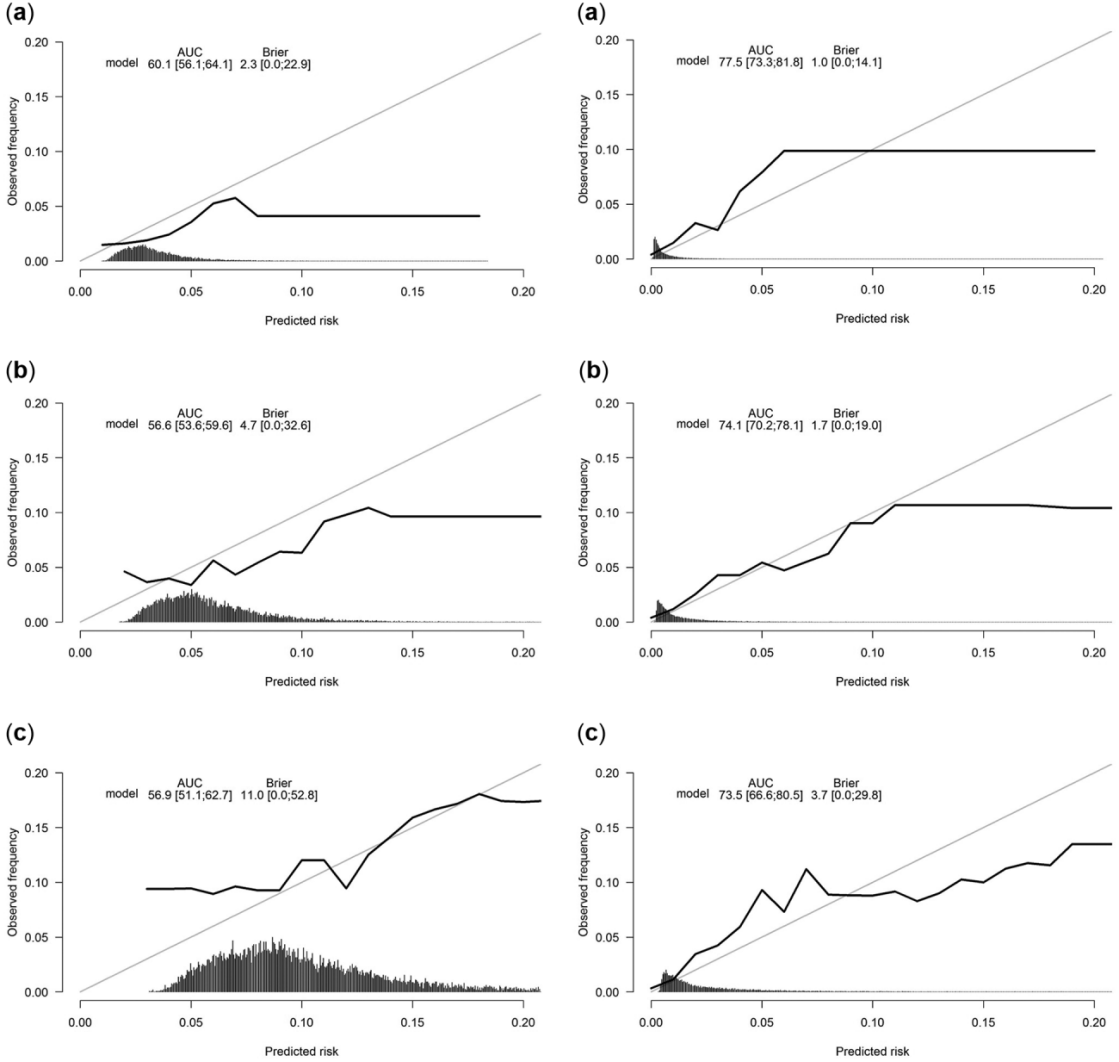
Calibration graphs modelling the risk of unemployment (left) and disability benefits (right) for 12 (a), 24 (b) and 60 (c) months of follow-up.

For unemployment, the C-index in the validation cohort was 0.60 (95% CI: 0.58–0.62) and an AUC of 0.57 (SE = 0.02) was found at 24 months of follow-up. For disability benefits, a C-index of 0.75 (95% CI: 0.73–0.77) was found and the AUC was 0.74 (SE = 0.02). Figure 1 shows the calibration graphs. Overall, calibration of the developed prediction models was reasonable. The sensitivity, specificity, the PPV and NPV were retrieved for 12, 24 and 60 months of follow-up (Supplementary table S4). For all models, the PPV was low (ranging from 5 to 19% for unemployment and from 0 to 18% for disability benefits) while the NPV was high (ranging from 89 to 98% for unemployment and from 97 to 100% for disability benefits).

## Discussion

Predictors for exit from paid employment through both unemployment and disability benefits were identified on the level of sociodemographic factors, chronic diseases, health behaviours and working conditions. Model performance in the development cohort and validation cohort yielded low C-indexes, but this improved for disability benefits when risk factors were modelled for workers with CVD, COPD or diabetes. The PPV of the models was low while the NPV of the models was high, indicating that the models more accurately predicted when workers remained employed rather than when workers exited paid employment.

The risk factors for involuntary exit from paid employment in the development cohort and the validation cohort correspond with risk factors found in previous research at the population level. Females are known to be at higher risk of early exit from paid employment.^29^ Furthermore, having a chronic disease was more strongly associated with disability benefits than with unemployment,^30^ which is a less health-driven pathway out of paid employment. Smoking and obesity have previously been shown to increase the risk of both involuntary exit routes.^5^^,^^31^ Lastly, the role of social support and low influence at work in involuntary exit through unemployment and disability benefits has also been found earlier.^32^^,^^33^ In the development cohort, having few possibilities for development was a predictor of both involuntary exit routes, and low meaning was a risk factor for unemployment. The Metlife Mature Market Institute (2006) in the USA indicated that, among older workers, an opportunity to do meaningful work was the primary reason to continue working.^34^ Whereas that study focused on retirement, results correspond with findings from the current study on unemployment.

Discriminative ability of the current model was lower compared with previous models for disability benefits in which moderate to strong C-indexes ranging from 0.70 to 0.85 were found.^13^^,^^14^ These differences can be explained by the fact that the previous studies included frequent work excusals or sick leave days in the past year,^13^^,^^14^ which are strongly related to subsequent disability benefits. When we restricted the study population to workers with specific chronic diseases, discriminative ability of the models increased for disability benefits. However, in previous studies as well as in the current study, the number of individuals who actually leave paid employment involuntary was low. This indicates that the model can better predict who will continue to work instead of who will exit paid work, as also shown in the high NPV and lower PPV. Therefore, it may be more suitable to examine the relative importance of these factors for early exit at a population level rather than making accurate predictions at the individual level.^15^ The current study has shed a light on which of these factors are important within these different disease groups. Working conditions seemed to be important especially for workers with COPD and diabetes, whereas smoking was especially important for workers with CVD and obesity was important for workers with rheumatoid arthritis. This information is relevant for occupational health care workers who can discuss these health behaviours and working conditions in consultations with workers.

A strength of the current study is the use of a large representative group of workers from the Lifelines Cohort Study. This enabled the stratification of models across specific chronic diseases, which were classified according to a combination of clinical measures, medication use and self-report. Furthermore, results were validated in a cohort among older workers in which similar constructs were measured. A limitation is different timing of the variables, as chronic diseases were measured at baseline, while health behaviours and working conditions were measured at wave 3. Additionally, the percentage of missing data was rather high for some variables, e.g. fruit and vegetable intake. With regard to the predictors, physical work demands—an important factor related to health-related job loss^35^—was unfortunately not measured in Lifelines. Another limitation was that the definition of specific chronic diseases differed between Lifelines and STREAM. Whereas workers with rheumatoid arthritis were included in Lifelines, workers with musculoskeletal problems were included in STREAM, which is a broader concept also including back and neck problems, resulting in a larger proportion of workers reporting this disease. Lastly, while Lifelines included workers of all ages, STREAM included older workers.

To conclude, sociodemographic factors, chronic diseases, unhealthy behaviours and working conditions were associated with unemployment and disability benefits. However, prediction models were not able to accurately estimate a personalized risk. Additional predictors are needed to improve the discriminative ability of prediction models. In addition, further research is needed to identify which predictors are the best targets for prevention. When the risk of the predictors was modelled for chronic diseases individually, model performance increased and personalized estimations were more accurate. Taking workers’ particular disease into account may contribute to the prevention of early exit from work into disability benefits.

## Supplementary data

Supplementary data are available at EURPUB online.

## Data availability statement

Data are stored at Statistics Netherlands. Data are available upon reasonable request, following the guidelines of the Statistics Netherlands.

## Supplementary Material

ckac045_Supplementary_Data
