# Supplementary material for: Development and validation of a prediction model for unemployment and work disability among 55 950 Dutch workers
Source: Eur J Public Health. 2022 May 25;32(4):578–85. doi: 10.1093/eurpub/ckac045 (PMC9341844; doi:10.1093/eurpub/ckac045)
Supplement: ckac045_Supplementary_Data [file ckac045_supplementary_data.zip › ejph-2021-06-om-0740-File003.docx]

**SUPPLEMENTARY FILE A**


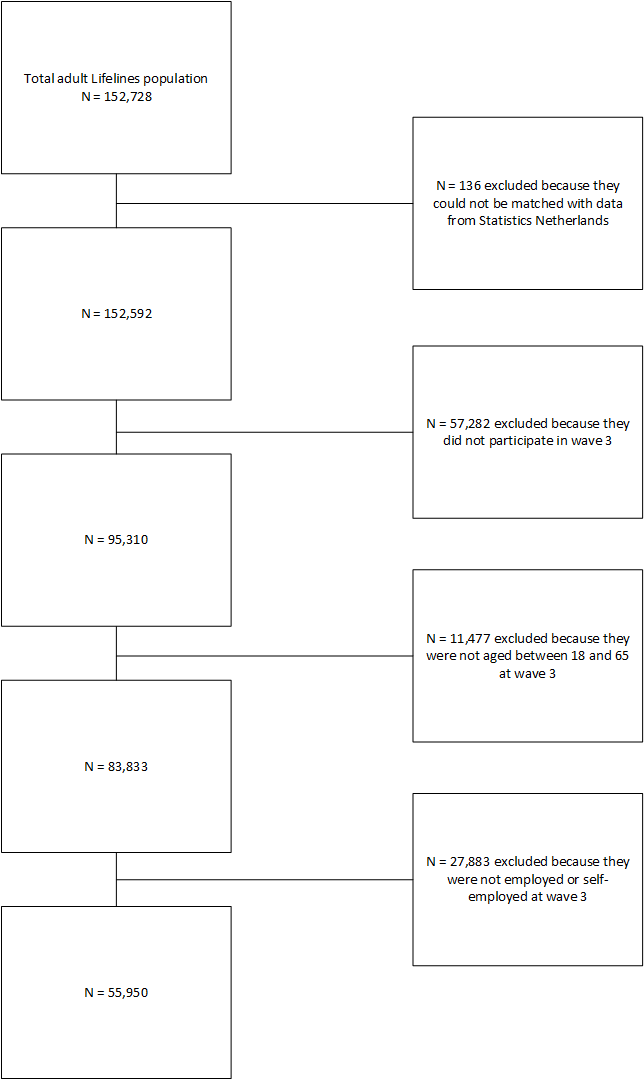


**Figure 1S.** Flow chart of inclusion in the development cohort (Lifelines Cohort Study).

**SUPPLEMENTARY FILE B**

**STREAM – validation cohort**

The external validation was conducted within the Dutch longitudinal Study on Transitions in Employment, Ability and Motivation (STREAM), using the first measurement wave from 2010. Recruitment took place through the Intomart GfK Online Panel, newsletters, banners, self-registration. Participants aged 45–64 years annually filled in an online questionnaire on a variety of topics, including sociodemographic factors, work characteristics and health (22). The Medical Ethical Committee of the VU University Medical Center Amsterdam declared that the Medical Research Involving Human Subjects Act does not apply to STREAM.

**Outcome variable**

Involuntary exit from paid employment was assessed in the same way as in the development cohort Lifelines using information from Statistics Netherlands which was linked to STREAM respondents (23).

**Predictors**Predictors of STREAM were selected to be in line with the prediction variables within Lifelines. Education was measured with a question on the highest level of education completed, and categorized into low (primary school, lower and intermediate secondary school or lower vocational training), intermediate (higher secondary school or intermediate vocational training) and high (higher vocational education or university education). Marital status was used to categorise individuals into those living together with a spouse or partner in the same household and others (i.e. those living alone).

*Sociodemographic factors*

Demographic variables included age, gender, educational level and marital status were included.

*Chronic disease & multimorbidity*

The presence of a chronic health problem was assessed using the following question: ‘Do you (currently) have one or more of the following chronic diseases, disorders or handicaps?’. Five diseases that closely resembled the chronic diseases in Lifelines were included: cardiovascular disease, respiratory diseases, psychological disease, musculoskeletal disease, and diabetes mellitus. Respiratory diseases, psychological disease and musculoskeletal disease most closely resembled COPD, depression and rheumatoid arthritis, respectively, and were therefore included for validation purposes. Multimorbidity was defined as having at least two of these diseases.

*Working conditions*

Quantitative job demands, work pace, autonomy, possibilities for development, meaningful work and social support were included. Quantitative job demands included two items with regard to how much and how hectic an individual’s work is. Work pace included two items on how fast one had to work and whether one had to work extra hard. Influence, often referred to as ‘autonomy’ within STREAM, was measured with five items about making decisions, deciding the order and speed of conducting tasks, having to find solutions and being able to take time off. Items on quantitative demands, work pace, and influence at work (autonomy) were derived from the Job Content Questionnaire (JCQ) (38). Social support at work was assessed using the COPSOQ-questionnaire with four items on how often colleagues and superior are willing to help, support and listen to work-related problems (27). Possibilities for development were examined using items on about trying to learn new things at work, feeling confident to cope with changes at work with regard to knowledge and skills, searching for people to learn something from, and thinking about whether one is able to keep doing a good job in the future (39). Meaning of work was self-constructed and participant could indicate to what extent they agreed with the statement ‘Work provides meaning to your life’. All items were used a 5-points answer scale. All questions were scored on a five-point scale, ranging from 1 to 5. The answer categories of the reversed working conditions were recoded so that a higher score reflected poorer working conditions. The domain-scores for influence (autonomy), possibilities for development and meaningful work were multiplied by 0.4, 0.5 and 2, respectively, so that the range of the domain scores was comparable with the domain score used in Lifelines, which resulted in a range of 2-10.

*Health behavior*Moderate physical activity was measured with one question indicating how many days someone usually performed physical activity (at work or outside work) for at least 30 minutes per week.

Smoking was measured with one question ‘Do you smoke?’ with three answer categories ‘Yes (1)’, ‘No, but I used to smoke (2)’ and ‘No, I have never smoked (3)’ and was dichotomized into smoking (1) and not smoking (2 and 3). BMI was derived from the weight and height of participants and expressed in kg/m2. Participants were categorized as having a healthy weight (BMI ≥18.5 to ≤24.9), overweight (BMI ≥25.0 to ≤29.9), or obese (BMI ≥30.0). STREAM did not include information on diet.

**SUPPLEMENTARY FILE C**

**Table 1S.** Prevalence (n and %) of involuntary exit from paid employment within specific groups of chronic diseases

|  | Total | Unemployment | Disability benefits |
| --- | --- | --- | --- |
| CVD | 583 | 52 (8.9) | 24 (4.1) |
| COPD | 2085 | 206 (9.9) | 53 (2.5) |
| Depression | 1188 | 163 (13.7) | 64 (5.4) |
| Diabetes | 988 | 101 (10.2) | 36 (3.6) |
| Rheumatoid arthritis | 775 | 69 (8.9) | 46 (5.9) |

*Notes:* Abbreviations: CVD=cardiovascular disease; COPD=chronic obstructive pulmonary disease.

**SUPPLEMENTARY FILE D**

|  | Unemployment *C=0.62 (0.61;0.63)*  *R^2^=0.02* | Disability benefits *C=0.68 (0.65;0.69)  R^2^=0.02* |
| --- | --- | --- |
| **Sociodemographic factors** |  |  |
| Age (per 10 years) | 1.04 (1.01; 1.08) | 0.80 (0.75; 0.86) |
| Gender | 1.21 (1.13; 1.29) | 1.76 (1.52; 2.05) |
| Marital status (in a relationship) | 0.74 (0.69; 0.81) | 0.85 (0.71; 1.01)* |
| Educational level (high = ref) |  |  |
| intermediate | 1.02 (0.95; 1.10) | 1.27 (1.08; 1.50) |
| low | 1.27 (1.16; 1.38) | 1.78 (1.46; 2.17) |
| **Chronic disease** |  |  |
| CVD | 1.06 (0.80; 1.39) | 2.67 (1.76; 4.06) |
| COPD | 1.08 (0.93; 1.24) | 1.43 (1.08; 1.90) |
| Depression | 1.43 (1.22; 1.67) | 2.48 (1.92; 3.21) |
| Diabetes | 1.12 (0.92; 1.37) | 1.90 (1.34; 2.69) |
| Rheumatoid arthritis | 1.00 (0.78; 1.26) | 3.03 (2.25; 4.10) |
| **Health behaviors** |  |  |
| Physical activity (0-7 days) | 0.98 (0.96; 0.99) | 0.97 (0.94; 1.01) |
| Smoking | 1.25 (1.16; 1.34) | 1.35 (1.15; 1.57) |
| Fruit intake (high = ref) |  |  |
| intermediate | 1.02 (0.93; 1.11) | 0.89 (0.74; 1.07) |
| low | 1.05 (0.96; 1.15) | 0.94 (0.78; 1.13) |
| Vegetable intake |  |  |
| intermediate | 0.94 (0.87; 1.03) | 1.03 (0.83; 1.27) |
| low | 0.99 (0.90; 1.09) | 0.99 (0.80; 1.22) |
| BMI (healthy weight = ref) |  |  |
| overweight | 1.01 (0.94; 1.08) | 0.96 (0.82; 1.11) |
| obesity | 1.20 (1.10; 1.30) | 1.39 (1.16; 1.66) |
| **Working conditions** |  |  |
| Quantitative demands | 0.99 (0.97; 1.01) | 1.01 (0.96; 1.06) |
| Work pace | 1.00 (0.98; 1.02) | 1.02 (0.97; 1.07) |
| Possibilities for development | 1.05 (1.03; 1.08) | 1.07 (1.01; 1.13) |
| Meaning of work | 1.12 (1.09; 1.14) | 1.03 (0.98; 1.09) |
| Influence at work | 1.01 (0.99; 1.03) | 1.04 (0.99; 1.08) |
| Social support | 1.11 (1.09; 1.14) | 1.06 (1.01; 1.11) |

**Table 2S.** The influence of personal and work-related predictors on involuntary exit from paid employment in the development cohort Lifelines (complete models).

**SUPPLEMENTARY FILE E
Table 3S.** The influence of personal and work-related predictors on unemployment in the development cohort Lifelines.

|  | CVD *C=0.63 (0.59;0,67) R^2^=0.03* | COPD *C=0.63 (0.59;0.67)*  *R^2^=0.03* | Depression  *C=0.62 (0.58; 0.66)*  *R^2^=0.03* | Rheumatoid arthritis  *C=0.66 (0.60;0.72)*  *R^2^=0.04* | Diabetes *C=0.63 (0.57;0.69)*  *R^2^=0.03* |
| --- | --- | --- | --- | --- | --- |
| **Sociodemographic factors** |  |  |  |  |  |
| Age (per 10 years) | 0.94 (0.60; 1.45) | 1.41 (1.08; 1.85) | 0.89 (0.75; 1.05) | 1.14 (0.84; 1.55) | 1.20 (0.88; 1.64) |
| Gender | 0.61 (0.32; 1.17) | 1.05 (0.79; 1.40) | 0.82 (0.59; 1.14) | 1.18 (0.66; 2.11) | 0.84 (0.56; 1.27) |
| Marital status | 0.56 (0.27; 1.17) | 0.76 (0.52; 1.13) | 0.59 (0.42; 0.84) | 0.53 (0.27; 1.07) | 0.89 (0.51; 1.53) |
| Educational level (high = ref) |  |  |  |  |  |
| intermediate | 1.04 (0.46; 2.36) | 0.87 (0.59; 1.28) | 1.03 (0.67; 1.58) | 1.28 (0.61; 2.65) | 0.89 (0.50; 1.60) |
| low | 1.32 (0.58; 3.00) | 1.15 (0.78; 1.69) | 1.13 (0.72; 1.78) | 1.77 (0.82; 3.82) | 1.29 (0.74; 2.25) |
| **Multimorbidity** |  |  |  |  |  |
| **Health behaviors** |  |  |  |  |  |
| Physical activity (0-7 days) | 0.96 (0.83; 1.11) | 0.94 (0.87; 1.00) | 0.96 (0.89; 1.04) | 0.99 (0.88; 1.12) | 0.97 (0.87; 1.07) |
| Smoking | 0.96 (0.46; 2.03) | 1.48 (1.09; 2.00) | 1.41 (1.01; 1.96) | 1.62 (0.95; 1.33) | 1.09 (0.66; 1.81) |
| Fruit intake (high = ref) |  |  |  |  |  |
| intermediate |  |  |  |  |  |
| low |  |  |  |  |  |
| Vegetable intake |  |  |  |  |  |
| intermediate |  |  |  |  |  |
| low |  |  |  |  |  |
| BMI (healthy weight = ref) |  |  |  |  |  |
| overweight | 0.59 (0.30; 1.18) | 0.97 (0.71; 1.34) | 0.73 (0.51; 1.06) | 0.60 (0.34; 1.06) | 1.34 (0.68; 2.63) |
| obesity | 0.99 (0.47; 2.09) | 1.18 (0.80; 1.74) | 1.16 (0.78; 1.72) | 0.81 (0.44; 1.50) | 1.85 (0.96; 3.58) |
| **Working conditions (higher is worse)** |  |  |  |  |  |
| Quantitative demands |  |  |  |  |  |
| Work pace |  |  |  |  |  |
| Possibilities for development | 1.10 (0.90; 1.34) | 1.12 (1.01; 1.24) | 1.06 (0.95; 1.18) | 1.03 (0.84; 1.25) | 0.98 (0.85; 1.12) |
| Meaning of work | 1.02 (0.84; 1.23) | 1.04 (0.94; 1.16) | 1.06 (0.95; 1.18) | 1.13 (0.93; 1.36) | 1.16 (1.01; 1.35) |
| Influence at work |  |  |  |  |  |
| Social support | 1.03 (0.84; 1.25) | 1.06 (0.96; 1.17) | 1.08 (0.97; 1.01) | 1.12 (0.95; 1.33) | 1.08 (0.95; 1.24) |

**SUPPLEMENTARY FILE F**

**Table 4Sa.** Sensitivity, specificity, positive predictive value (PPV), and negative predictive value (NPV) for unemployment.

| Unemployment |  | Threshold (predicted values) |  |  |
| --- | --- | --- | --- | --- |
|  |  | 0.05 | 0.1 | 0.2 |
| Time |  |  |  |  |
| 12 months | Sens | 0.21 | 0.01 | - |
|  | Spec | 0.90 | 1.00 | - |
|  | PPV | 0.05 | 0.07 | - |
|  | NPV | 0.98 | 0.98 | - |
| 24 months | Sens | 0.65 | 0.12 | 0.00 |
|  | Spec | 0.44 | 0.94 | 1.00 |
|  | PPV | 0.05 | 0.09 | 0.06 |
|  | NPV | 0.96 | 0.96 | 0.95 |
| 60 months | Sens | 0.05 | 0.05 | 0.05 |
|  | Spec | 0.97 | 0.98 | 0.98 |
|  | PPV | 0.11 | 0.19 | 0.19 |
|  | NPV | 0.92 | 0.89 | 0.89 |

**Table 4Sb.** Sensitivity, specificity, positive predictive value (PPV), and negative predictive value (NPV) for disability benefits.

| Disability benefits |  | Threshold (predicted values) |  |  |
| --- | --- | --- | --- | --- |
|  |  | 0.05 | 0.1 | 0.2 |
| Time |  |  |  |  |
| 12 months | Sens | 0.06 | 0.02 | 0.00 |
|  | Spec | 1.00 | 1.00 | 1.00 |
|  | PPV | 0.11 | 0.18 | 0.00 |
|  | NPV | 0.99 | 0.99 | 1.00 |
| 24 months | Sens | 0.16 | 0.05 | 0.01 |
|  | Spec | 0.97 | 0.99 | 1.00 |
|  | PPV | 0.07 | 0.13 | 0.18 |
|  | NPV | 0.99 | 0.98 | 0.98 |
| 60 months | Sens | 0.34 | 0.10 | 0.02 |
|  | Spec | 0.88 | 0.97 | 1.00 |
|  | PPV | 0.09 | 0.10 | 0.18 |
|  | NPV | 0.97 | 0.97 | 0.97 |
